# Clinical Experience of Patients Referred to a Multidisciplinary Cardiac Oncology Clinic: An Observational Study

**DOI:** 10.1155/2015/671232

**Published:** 2015-08-02

**Authors:** Jeffrey Sulpher, Shrey Mathur, Nadine Graham, Freya Crawley, Michele Turek, Christopher Johnson, Ellamae Stadnick, Angeline Law, Jason Wentzell, Susan Dent

**Affiliations:** ^1^The Ottawa Hospital Cancer Centre, University of Ottawa, Ottawa, ON, Canada K1H 8L6; ^2^Division of Cardiology, The Ottawa Hospital, University of Ottawa, Ottawa, ON, Canada K1H 8L6; ^3^Department of Pharmacy, The Ottawa Hospital, Ottawa, ON, Canada K1H 8L6

## Abstract

Cardiotoxicity is the second leading cause of long-term morbidity and mortality among cancer survivors. The purpose of this retrospective observational study is to report on the clinical and cardiac outcomes in patients with early stage and advanced cancer who were referred to our multidisciplinary cardiac oncology clinic (COC). A total of 428 patients were referred to the COC between October 2008 and January 2013. The median age of patients at time of cancer diagnosis was 60. Almost half of patients who received cancer therapy received first-line chemotherapy alone (169, 41.7%), of which 84 (49.7%) were exposed to anthracyclines. The most common reasons for referral to the cardiac oncology clinic were decreased LVEF (34.6%), prechemotherapy assessment (11.9%), and arrhythmia (8.4%). A total of 175 (40.9%) patients referred to the COC were treated with cardiac medications. The majority (331, 77.3%) of patients were alive as of January 2013, and 93 (21.7%) patients were deceased. Through regular review of cardiac oncology clinic referral patterns, management plans, and patient outcomes, we aim to continuously improve delivery of cardiac care to our patient population and optimize cardiac health.

## 1. Introduction

With the evolution of systemic and targeted therapies in cancer treatment, it has become increasingly evident that damage to the heart may occur as a result of cancer therapy. While cancer survivorship has significantly increased over the last decade [1], cardiotoxicity is the second leading cause of long-term morbidity and mortality among cancer survivors [2]. In addition, there are an increasing number of cancer patients with preexisting heart disease, for whom treatment with potentially cardiotoxic cancer therapy may pose a challenge [1]. Prevention and management strategies of cardiotoxicity will be important to optimize cancer care while maintaining cardiovascular health. Hence, the need for collaboration between oncologists and cardiologists from diagnosis to survivorship is imperative to ensure patients are receiving the best possible cancer care.

Modern cancer therapies can be complex, and their potential impact on cardiovascular health may compromise the provision of the best available cancer treatment. For patients and their families, receiving a cancer diagnosis and navigating the cancer care system poses a significant challenge. These difficulties may be compounded if cardiac complications arise from cancer therapy, and multiple medical specialties are involved in the patient's circle of care. Historically, cancer patients experiencing cardiotoxicity related to their cancer treatment have been referred to cardiologists with minimal knowledge of the importance of these cancer therapies and their impact on cardiovascular health [3]. This has led to significant variability in the assessment and management of these patients.

Although cardiotoxicities associated with conventional chemotherapy are well known, the short- and long-term effects of targeted agents on the heart are less well understood. A growing number of targeted therapies (e.g., mTOR inhibitors, tyrosine kinase inhibitors, and VEGF inhibitors), given as single agents or in combination with systemic therapy, are being approved for use in a wide variety of malignancies. For example, agents that target angiogenesis via inhibition of vascular endothelial growth factor receptor pathways (e.g., bevacizumab, sunitinib, and sorafenib) have been shown to improve survival in patients with several solid tumours, including colorectal, renal, and hepatocellular carcinomas [4, 5]. However, the potential impact of these agents on the cardiovascular health of cancer survivors (e.g., congestive heart failure, hypertension) is less clear [6, 7]. A recent meta-analysis of 7000 patients treated with the tyrosine kinase inhibitor sunitinib demonstrated a 4.1% incidence of treatment-related heart failure [8]. A similar analysis of 900 patients treated with sorafenib observed a 1% rate of cardiac dysfunction [9]. Due to the retrospective nature of these data, more studies are required to establish a direct link, as well as investigation of other indirect effects and toxicities seen in this patient population [10].

In order to provide cancer patients with the best possible therapy without compromising cardiac health, a multidisciplinary (medical oncology, cardiology, pharmacy, and nursing) cardiac oncology clinic was established at The Ottawa Hospital in 2008—the first program of its kind in Canada [11]. The goals of the cardiac oncology clinic are to streamline referral of patients with cardiac complications related to cancer therapies; gain expertise in the management of cancer therapy–induced cardiotoxicity; provide consistent cardiac care; and further the cardiac oncology field through research and education.

The purpose of this retrospective observational study is to report on the clinical and cardiac outcomes of patients with early stage and advanced cancer who were referred to our multidisciplinary cardiac oncology clinic. This study was approved by the Ottawa Hospital Research Ethics Board.

## 2. Patients and Methods

All cancer patients (early and advanced stage) treated at the Ottawa Hospital Cancer Center and referred to the cardiac oncology clinic between October 2008 and January 2013 were eligible for this retrospective observational study. Data collection included patient demographics, cardiac risk factors, cancer treatment and completion rates, cardiac assessments (echocardiogram/MUGA) prior to and during cancer treatments, cardiac treatment, and clinical outcomes (disease progression, death). Data on cancer radiation treatments was not collected. Patients were referred to the cardiac oncology clinic by their primary oncologist if they had a LVEF <50% presystemic therapy, a decline in LVEF to <50% during treatment, a decline in LVEF by ≥10 percentage points during treatment, concerns regarding treatment-related cardiotoxicity, symptoms of other cardiac diseases (e.g., arrhythmia, pericardial disease, coronary artery disease, and valvulopathy), or evidence of symptomatic congestive heart failure. Changes in LVEF were calculated based on percentage differences from baseline assessment. MUGA scans and echocardiograms (to assess LVEF) were done in the majority of patients prior to commencing cancer therapy and as per institution policy. Additional cardiac investigations were performed at the discretion of the treating physician.

## 3. Results

Between October 2008 and January 2013, 428 patients were referred to the cardiac oncology clinic. Baseline patient demographics are shown in Table 1. The median age of patients at the time of cancer diagnosis was 60 years (r: 18–90 years). The majority of patients had breast cancer (246, 57.5%), followed by gastrointestinal malignancies (63, 14.7%) and genitourinary malignancies (52, 12.1%). Less common tumour types included lung, sarcoma, thyroid, and haematological malignancies. Patients had a median of two (r: 0–7) cardiovascular risk factors at the time of referral to the cardiac oncology clinic, the most common risk factors being smoking (188, 43.9%), hypercholesterolemia (173, 40.4%), obesity (123, 28.7%), and hypertension (114, 26.6%).

The majority (405, 94.6%) of patients received cancer therapy as outlined in Table 2. First-line therapy included chemotherapy alone (169, 41.7%), targeted therapy alone (24, 5.9%; monoclonal antibodies or tyrosine kinase inhibitors), and combined therapy (163, 40.2%). Of those who received first-line chemotherapy alone, 84 (49.7%) patients were exposed to anthracycline-based regimens. The median anthracycline exposure was 277 mg/m^2^ (r: 46–4803 mg/m^2^). The median number of first-line chemotherapy cycles was 6 (r: 0–59 cycles), and 128 (31.6%) patients received second-line systemic therapy (median number of cycles = 5.5 (r: 0–33 cycles)).

The most common reasons for referral to the cardiac oncology clinic were decreased left ventricular ejection fraction (LVEF) (148, 34.6%), prechemotherapy assessment (51, 11.9%), and arrhythmia (36, 8.4%). Less common reasons included angina, congestive heart failure, and cardiomyopathy (Table 3).

Chemotherapy outcomes are illustrated in Table 4. As of January 2013, the majority (224, 60.4%) of patients who received chemotherapy (341, 79.7%) successfully completed their recommended therapy. A further twelve patients (3.5%) were receiving ongoing treatment. Reasons for discontinuation of chemotherapy (105, 30.8%) varied considerably, but the majority were due to change in clinical status (e.g., disease progression). Eighty-four (19.6%) patients were receiving ongoing targeted therapy or other medications; outcome data was not available for these patients.

**Figure 1:**
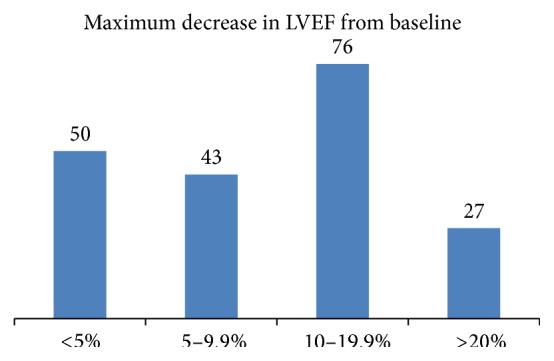
Maximum decrease in LVEF from baseline (*N* = 196).

Cardiac outcomes are illustrated in Table 5. The majority (381, 89.0%) of patients had baseline cardiac imaging (MUGA/echocardiogram) performed prior to commencing cancer treatment. Subsequent cardiac imaging was performed at the discretion of the treating physician. A large number of patients (196, 51.4%) exhibited at least one episode of decreased LVEF from baseline. The majority (76, 38.8%) of these patients exhibited a decrease between 10% and 19.9% and 27 (13.8%) patients had a decrease in LVEF of more than 20% (Figure 1). Recovery of LVEF to baseline was seen in 55 (28.0%) patients and partial recovery was recorded in a further 16 (8.2%) patients. However, further decline in LVEF occurred in 55 (28.0%) patients. A total of 59 (30.0%) patients achieved stable LVEF with cardiac intervention. A total of 175 (40.9%) patients referred to the cardiac oncology clinic were treated with cardiac medications; 39 (22.3%) were treated with ACE inhibitors, 22 (12.6%) were treated with beta-blockers, and 24 (13.7%) were treated with both. Multiple medications were prescribed for 90 (51.4%) patients.

Overall patient outcomes are described in Table 6. The majority (331, 77.3%) of patients were alive as of January 2013, and 93 (21.7%) patients were deceased. The majority of deaths were due to cancer progression (81, 87.1%), followed by cardiac etiologies (6, 6.4%) and other causes (6, 6.4%). Patient outcomes are unknown for 4 (0.9%) patients due to incomplete follow-up.

## 4. Discussion

The evolution of personalized medicine has led to an increasing interest in the development and testing of targeted therapies in oncology. While cancer professionals have been well versed in the identification and treatment of toxicities associated with chemotherapy, there is less understanding of the short- and long-term consequences associated with targeted agents. The United Kingdom National Cancer Research Institute (NCRI) has recently published trastuzumab cardiac toxicity monitoring guidelines based on data obtained from original clinical trials [12]. In this retrospective observational study, we report on the clinical outcomes of cancer patients treated with chemotherapy and/or targeted agents who were referred to our dedicated cardiac oncology clinic. While the majority of referrals to our clinic were breast cancer patients with decreased LVEF, 40% were patients with other tumour types (gastrointestinal, genitourinary, and hematologic) with a wide variety of cardiovascular issues including coronary artery disease, arrhythmias, and angina. The majority of patients were able to complete their prescribed cancer therapy (224, 60.4%), of which 191 (85.3%) did so during or after completing cardiac therapy. In cases where a change in therapy was not required, the cardiac oncology assessment often resulted in reassurance to both the patient and the referring physician. A total of 105 (30.8%) patients discontinued cancer treatment earlier than planned, mainly due to disease progression. These findings are consistent with an earlier review of our clinical data, specifically in the breast cancer population [11].

In this study, data was collected retrospectively from a variety of sources including hospital records (e-charts and paper charts) and nonhospital based imaging centers. We included all cancer patients referred to our clinic (including early and late stage disease), thus making conclusions about clinical outcomes difficult in such a heterogeneous population. We did not collect data on radiation therapy in our early patient database; future registries should also include this information. Our data would suggest that with appropriate cardiac management, many cancer patients could complete their prescribed therapy; however a case-cohort study would provide stronger evidence to support this statement.

Since the inception of our clinic, a number of similar cardiac oncology clinics have been introduced across North America. To our knowledge, this study is the first to describe a patient population specifically referred for cardiac oncology care and to characterize clinical outcomes. If we are to advance patient care and the growing field of cardiac oncology, it is imperative that we collaborate with our multidisciplinary colleagues. The National Cancer Institute (NCI) and National Heart, Lung and Blood Institute (NHLBI) recently sponsored a two-day workshop to identify the knowledge gaps in the field of cardiac oncology. Highlights of the workshop recommendations included the promotion of clinical research in the early identification and treatment of cardiotoxicity, as well as the need to identify the long-term cardiac consequences of these therapies in cancer survivors [13]. In conjunction with the Canadian Cardiac Oncology Network (CCON), we are in the process of formulating a national prospective clinical cardiac oncology registry, which will capture clinical data in real time, and allow sharing of data with other cardiac oncology clinics throughout Canada and eventually North America.

In summary, while cancer therapy continues to improve patient outcomes, the risk of unintended toxicities, such as cardiotoxicity, remains a concern. Our dedicated cardiac oncology clinic aims to identify patients at risk of cancer therapy-related cardiac complications, so that these issues can be managed and long-term sequelae avoided. From the perspective of the referring oncologist, a dedicated cardiac oncology clinic provides reassurance with management of high risk patients and streamlines communication between specialties. Research efforts are underway to develop practical cardiac risk stratification tools, in order to select oncology patients who may benefit from more intensive cardiac monitoring during cancer therapy and follow-up. Through regular review of cardiac oncology clinic referral patterns, management plans, and patient outcomes, we aim to continuously improve the delivery of cardiac care to our patient population while optimizing cancer therapy and to conserve valuable resources by creating efficiencies within the health system.

## Figures and Tables

**Table 1:** Patient demographics (*N* = 428).

	*N* (%)
Median age at diagnosis	60 years (r: 18–90 years)
Gender	
(i) Female	300 (70.1%)
(ii) Male	128 (29.9%)
Primary tumour type	
(i) Breast	246 (57.5%)
(ii) Gastrointestinal	63 (14.7%)
(iii) Genitourinary	52 (12.1%)
(iv) Haematological	31 (7.2%)
(v) Lung	17 (4.0%)
(vi) Other^*^	19 (4.4%)
Cardiac risk factors (median)	2 (r: 0–7)
(i) Smoker	188 (43.9%)
(ii) Hypercholesterolemia	173 (40.4%)
(iii) Obesity (BMI > 30)	123 (28.7%)
(iv) Hypertension	114 (26.6%)
(v) Diabetes	57 (13.3%)
(vi) Coronary artery disease	21 (4.9%)

^∗^Other tumour sites: gynaecologic, skin, sarcoma, neurologic, amyloidosis, thyroid, musculoskeletal.

**Table 2:** Cancer therapy (*N* = 405).

	*N* (%)
Cancer therapy	*N* = 405
(i) First-line chemotherapy alone	169 (41.7%)
(ii) First-line targeted therapy alone^*^	24 (5.9%)
(iii) First-line combined therapy (chemotherapy and targeted therapy)	163 (40.2%)
(iv) Second-line therapy (chemotherapy and/or targeted therapy)	128 (31.6%)
First-line chemotherapy alone	*N* = 169
(i) Anthracycline-based	84 (49.7%)
(ii) Non-anthracycline-based	85 (50.3%)
(iii) Median anthracycline dose	277 mg/m^2^ (r: 46–4803)
Number of chemotherapy cycles (median)	
(i) First-line chemotherapy	6 (r: 0–59 cycles)
(ii) Second-line chemotherapy	5.5 (r: 0–33 cycles)

^∗^Targeted therapy examples: trastuzumab, sunitinib, bevacizumab, sorafenib, and imatinib.

**Table 3:** Reason for referral to the cardiac oncology clinic (*N* = 428).

	*N* (%)
Reasons for referral	*N* = 428
(i) Decreased LVEF	148 (34.6%)
(ii) Prechemotherapy assessment	51 (11.9%)
(iii) Arrhythmia	36 (8.4%)
(iv) Congestive heart failure	24 (5.6%)
(v) Cardiomyopathy	14 (3.3%)
(vi) Other^*^	128 (29.9%)

^∗^Other examples: pericardial disease, valvular heart disease, coronary artery disease, and hypertension.

**Table 4:** Chemotherapy outcomes (*N* = 341).

	*N* (%)
Completed chemotherapy	*N* = 224 (60.4%)
(i) Prior to beginning cardiac therapy	33 (14.7%)
(ii) During cardiac therapy	56 (25%)
(iii) After completing cardiac therapy	135 (60.2%)
Resumed/ongoing	12 (3.5%)
Discontinued	105 (30.8%)

**Table 5:** Cardiac outcomes (*N* = 428).

	*N* (%)
Mode of prechemotherapy LVEF assessment	*N* = 381
(i) ECHO	286 (5.1%)
(ii) MUGA	84 (22.0%)
(iii) Other/combined modalities	11 (2.9%)
(iv) Pre-chemo-LVEF (median)	60% (r: 25.0–81.2)
Change in LVEF	*N* = 381
(i) No significant decline	232 (60.9%)
(ii) Any decline	196 (51.4%)
LVEF outcome	N = 196
(i) Full recovery	55 (28.0%)
(ii) Partial recovery	16 (8.2%)
(iii) Stable	59 (30.0%)
(iv) Progressive decline	55 (28.0%)
(v) Unknown	11 (5.8%)
Cardiac medication(s)	*N* = 175
(i) ACE inhibitors	39 (22.3%)
(ii) Beta-blockers	22 (12.6%)
(iii) ACE inhibitors + beta-blockers	24 (13.7%)
(iv) Multiple	90 (51.4%)

**Table 6:** Patient outcomes (*N* = 428).

	*N* (%)
Living	331 (77.3%)
Deceased	*N* = 93 (21.7%)
(i) Progression	81 (87.1%)
(ii) Cardiac etiologies	6 (6.4%)
(iii) Other	6 (6.4%)
Lost to follow-up	4 (0.9%)
